# Short learning curve associated with robotic total knee arthroplasty: A retrospective study

**DOI:** 10.1002/jeo2.70401

**Published:** 2025-09-04

**Authors:** Alexandre Le Guen, Antoine Mouton, Guillaume Auberger, Vincent Le Strat, Simon Marmor, Thomas Aubert

**Affiliations:** ^1^ Department of Orthopaedic Surgery Groupe Hospitalier Diaconesses Croix Saint‐Simon Paris France

**Keywords:** learning curve, surgical robotics, total knee arthroplasty

## Abstract

**Purpose:**

Robotic‐assisted total knee arthroplasty (RA‐TKA), which is increasingly used to improve surgical precision, can face adoption difficulties due to a learning curve marked by longer operating times. The aim of this study was to evaluate the learning curve associated with the VELYS™ robot in five surgeons from the same centre with different annual arthroplasty volumes using navigated assistance with personalised alignment. The primary aim was to assess the learning curve for each surgeon. Secondary aims were to identify the factors associated with extended operative times.

**Methods:**

In this retrospective comparative study, 367 patients who underwent primary TKA between January and December 2024 were included, comprising 149 with robotic assistance and 218 with navigated assistance. The surgical learning curve, based on skin‐to‐skin operating time, was assessed using the cumulative summation method. Five surgeons were evaluated: two high‐volume surgeons (>150 TKAs per year), a medium‐volume surgeon (between 50 and 150) and two low‐volume surgeons (<50). Pre‐ and intra‐operative data (age, gender, body mass index, American Society of Anesthesiologists score, pre‐operative hip–knee–ankle, range of motion, approach, size and implant constraint and type of assistance) were collected to identify extended operative time factors.

**Results:**

The learning curve was reached after performing between 4 and 11 cases (11 procedures for surgeon no. 1, 4 for surgeon no. 2, 6 for surgeon no. 3, 4 for surgeon no. 4 and 4 for surgeon no. 5). The robotic operating time was 57.1 min compared to 54.1 min (*p* = 0.017) with navigation. The increase was statistically significant only for one low‐volume surgeon (*p* = 0.008). Use of the robot (*p* < 0.001), surgeon (*p* < 0.001), use of a posterior‐stabilised implant (*p* < 0.001) and varus of more than 10° (*p* = 0.0191) were independent factors associated with extended operative time.

**Conclusion:**

The learning curve associated with VELYS™ was between 4 and 11 procedures. The small increase in operative time compared to navigation should not be a barrier to its adoption.

**Level of Evidence:**

Level III, case–control retrospective analysis.

**Abbreviations**
RA‐TKA: robotic‐assisted total knee arthroplasty
TKA: total knee arthroplasty

## INTRODUCTION

Total knee arthroplasty (TKA) is one of the most commonly performed procedures in orthopaedics, and its volume is expected to continue to grow dramatically [24]. Recently, the use of robotic‐assisted TKA (RA‐TKA) is gaining traction as a potential means of reducing complications and optimising the accuracy of implant positioning [6, 7, 13, 14, 32]. Nevertheless, the implementation of robotics in a centre may be limited by its specific complications linked to technology‐related failures [30] or pin tracts [25, 28]. In addition, the learning curve, which manifests itself in extended operative times, could limit surgeons from adopting robotic‐assisted surgery as it could impact patient outcomes, increasing the risk of infections and straining hospital workflows through reduced operating room efficiency and higher costs [2, 3, 16].

Over the past decade, equipment manufacturers have introduced several robotic systems with both semi‐autonomous and autonomous design platforms [11]. Among these, the VELYS™ Robotic‐Assisted Solution is an imageless system that does not require preoperative Computed Tomography scans [11]. It uses optical tracking technology and bone‐mounted arrays to perform bone resections with a bed‐mounted robotic‐assisted system that guides the surgical saw in space [7]. Previous studies showed that the learning curve depends on the type of assistance used, and only a few have assessed the VELYS™ [8, 16, 18, 30]. Most of these studies focused on the training of a single surgeon with no navigation experience [12, 16, 18].

In this study, we aimed to assess the learning curve associated with VELYS™ Robotic‐Assisted Solution for five surgeons with different volumes of annual arthroplasties from a single centre, with the same nurse team, transitioning from navigated instrumentation with personalised alignment to robotic instrumentation with the same alignment philosophy.

We hypothesised that the learning curve for RA‐TKA using the VELYS™ system would be relatively short.

The primary aim of this study was (1) to investigate the learning curve for revision TKA (rTKA) in relation to surgical time. The secondary aim was (2) to identify the associated factors for extended operative times.

## MATERIALS AND METHODS

### Data collection

A single‐centre retrospective consecutive cohort study was conducted. This study was approved by the local institutional review board (CNIL MR004 2225508) and complied with the principles outlined in the Declaration of Helsinki.

Patients who underwent a TKA using the ATTUNE™ implant (Johnson & Johnson), with either robotic or navigated assistance, in the Department of Orthopedic Surgery at Diaconesses Croix Saint Simon Hospital in Paris, between 1 January and 31 December 2024, were reviewed for inclusion. Patients undergoing unicompartmental arthroplasties or revision total knee arthroplasties were not eligible for inclusion in this study. All arthroplasties were performed without the use of a tourniquet, and cemented fixed‐bearing primary total knee implants were used in all cases. Surgery was partly performed by a resident, and patients who had undergone rTKA or unicompartimental arthroplasty were excluded. A formal sample size calculation was not performed for the learning curve analysis, as the objective was to explore operative time trends over consecutive cases for each surgeon.

**Figure 1:**
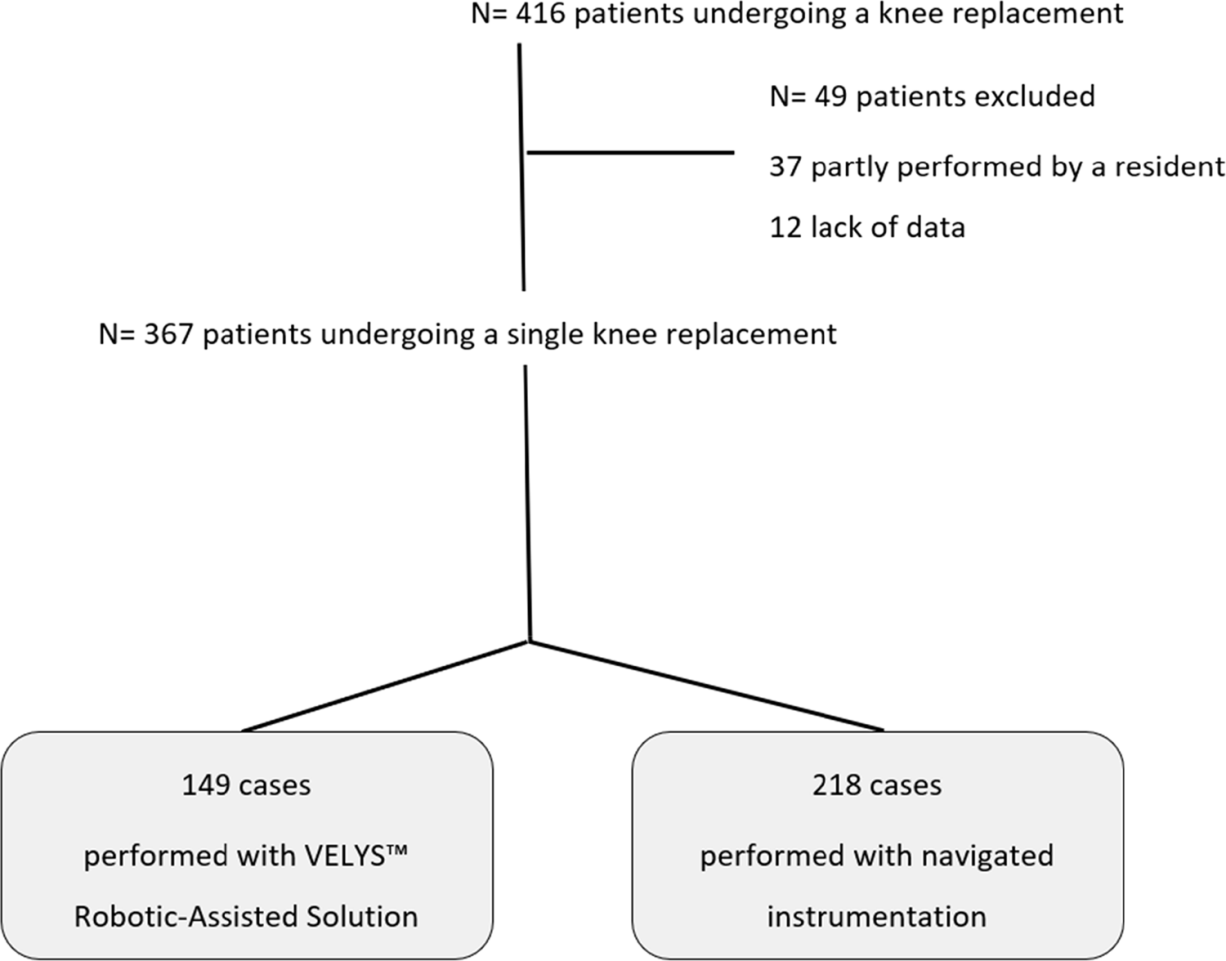
Flow diagram of patient selection for the analyses.

Of the 413 eligible patients, 49 patients were excluded: 37 patients were partly performed by a resident, and 12 cases were excluded due to data collection errors caused by the stopwatch being inadvertently left running. Of the 367 included patients, 149 were performed with VELYS™ Robotic‐Assisted Solution (Johnson & Johnson, Medtech) and 218 were performed with navigated instrumentation (Brainlab Knee3 Navigation) (Figure 1).

Patients undergoing navigation‐assisted TKA were included to serve as a comparator group, as this technique represented the previous gold standard in this centre. Including this cohort allowed us to contextualise the surgical times associated with RA‐TKA by comparing them to those of the established navigation‐assisted approach, thereby providing a meaningful reference for evaluating the learning curve of the robotic system.

Five surgeons were evaluated, whose number corresponds to the order of use of the robot from June to September 2024: two high‐volume surgeons with more than 150 TKA per year (numbers 1 and 2), one medium‐volume surgeons with a volume between 50 and 150 (number 5) and two low‐volume surgeons with an annual volume less than 50 arthroplasties (numbers 3 and 4). Training for the RA‐TKA procedure was facilitated through hands‐on sessions in a mobile laboratory, where the surgeon practised on a surgical simulator. A representative from the company (Johnson and Johnson, Medtech) was present during each surgeon's first cases.

All patients underwent a clinical examination by a knee surgeon and had a knee X‐ray consistent with a diagnosis of symptomatic knee osteoarthritis. Limb alignment (hip–knee–ankle [HKA] angle) was measured on full‐length x‐rays based on a standardised protocol [23]. The preoperative clinical examination included a standard knee examination with the knee range of motion (recurvatum–flexion contracture–maximal flexion).

Clinical information, including age, gender, body mass index (BMI) and American Society of Anesthesiologists (ASA) score [21], was collected.

Intra‐operatively, the surgical time from skin to skin was timed. Approach (transquadricipital, lateral, midvastus or subvastus), size of implants and constraint of the prosthesis (cruciate‐retaining or posterior‐stabilised) were recorded.

### Personalised alignment philosophy

Each knee exhibits a unique combination of constitutional alignment, degenerative deformity and individual ligamentous laxity. All implants were balanced using a patient‐specific alignment strategy aimed at restoring the patient's native soft tissue laxity, without the need for any ligament releases [31].

Deformities measuring less than 10° were corrected to a mechanical axis between 0° and 3°, while more severe deformities (>10°) were corrected to a range between 0° and 5°. Preoperative varus knees were intentionally corrected to a postoperative HKA angle of less than 180°, while valgus knees were adjusted to remain within a targeted safe zone, with HKA angles ranging from 177° to 183° [9].

First, medio‐lateral balancing in extension is targeted using a tibial cut obliquity between 3° of valgus and 3° of varus and the distal femoral cut obliquity ranged from 5° of valgus to 5° of varus, depending on the coronal plane deformity. At full knee extension, the difference between lateral and medial laxity measurements was aimed to be <1 mm [1].

Then, medio‐lateral balancing in flexion is managed by the obliquity of the posterior femoral cut, which modifies the rotation of the femoral component. We restricted this rotation between 6° of external rotation and 2° of internal rotation. At 90° of knee flexion, the difference between lateral and medial laxity measurements was aimed to be <2 mm [1].

Finally, balancing of the extension–flexion spaces is managed by adjusting the thickness of the tibial or distal femoral cuts, the flexion of the femoral component, or its anteroposterior positioning. From full extension to 90° of knee flexion, the difference between medial laxity measurements was aimed to be <1 mm [1].

The tibial cut can be increased in cases of significant tibial wear or decreased in cases of excessive resection (9 mm on the less‐used compartment), or in cases of genu valgum (under‐resected to 7 mm).

The thickness variation of the distal femoral cut was restricted to ±2 mm to minimise patellofemoral stress during flexion and reduce the risk of mid‐flexion instability.

Finally, adjusting the flexion angle of the femoral component between 3° and 6° allows us to reduce the flexion gap and the risk of notching.

The anteroposterior positioning of the femoral implant was optimised to fine‐tune the flexion gap while avoiding anterior overhang and posterior notching.

### Robotic surgical set‐up

**Figure 2:**
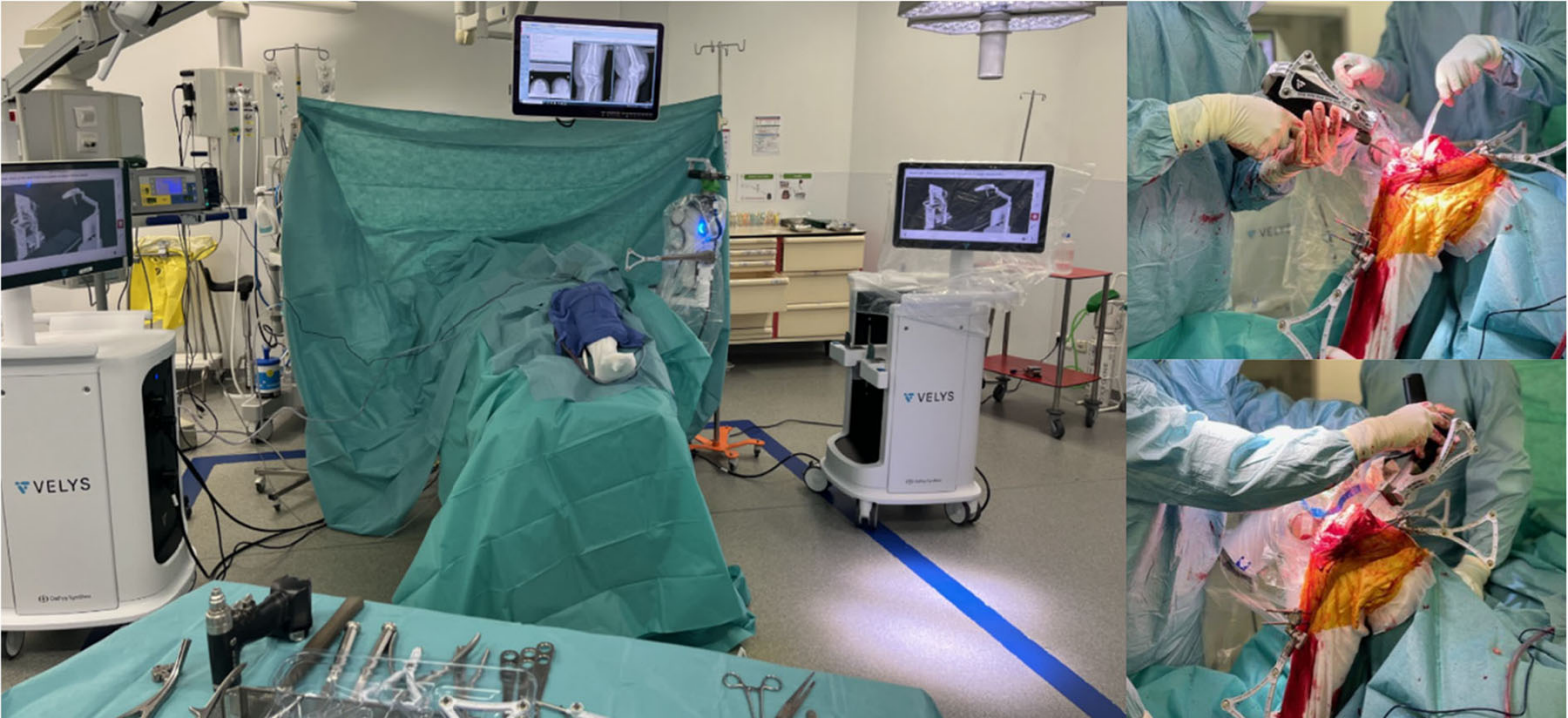
Robotic surgical set‐up.

Patients were positioned supine on the operating table with lateral femoral support and bolster to facilitate flexion and extension of the leg. As shown in Figure 2, the robot is positioned on the operator side.

Proximal tibial and distal femoral registration pins were inserted adjacent to the tibial tubercle and medial epicondyle, respectively. Tibial arrays were placed outside the incision, while femoral arrays were placed within the incision via self‐drilling and self‐tapping pins. The optical arrays were then mounted. The articulating robotic arm was secured to the operating table. Bone morphing registration and peroperative laximetry, exerting forced varus and forced valgus from extension to maximum flexion, were performed to proceed to the gap balancing. Limb alignment and knee balance were optimised using a range of motion with provisional implants on the planning screen. The robotic arm was then used to guide bone resections of the femur and tibia according to the plan. All patients were treated with tibia‐first gap‐balanced personalised alignment technique. Trial implants were placed, and the range of motion, flexion and extension gaps, limb alignment and patellar tracking were assessed.

### Navigation set‐up

In a similar way, navigation pins were fixed by drilling the distal femur and the proximal tibia [19, 20]. The system is based on real‐time optical tracking technology, utilising a stereoscopic infra‐red camera and instruments equipped with reflective markers. Based on the intraoperative registration of specific anatomical and peroperative laximetry, the Brainlab Knee3 navigation system (Brainlab) allows the surgeon to place guides on the femur and the tibia and to verify the cut effect in balancing flexion and extension gaps [19].

### Statistical analyses

Before analysis, verification was conducted to identify missing, aberrant or inconsistent data. After corrections, the database was locked. Analysis was performed on the locked database.

For the primary outcome measure, the learning curve cumulative summation (CUSUM) analysis was used to evaluate RA‐TKA surgical times and infer changes in performance over time. This statistical tool quantifies the running total of differences between individual surgical times and the mean of all surgical times to assess the change in the surgical times. Any specific CUSUM value represents the sum of all CUSUM values up to that point.

The CUSUM values were then plotted against the chronologically ordered procedures. Two distinct phases constitute the learning curve: (1) the initial learning curve and (2) the plateau of the learning curve or period of increased competence. Breakpoints in the data were identified by applying a piecewise linear regression model to distinguish the individual phases of the learning curve. In addition, the case number at which the CUSUM value entered the plateau was identified as the number of cases to proficiency. This phase represents the stabilisation of operating times and the approximation of the mean operating time value.

For the secondary outcome, patient characteristics were summarised using descriptive statistics appropriate to the nature of each variable. Descriptive statistics included mean with standard deviation (SD) for continuous variables and number of non‐missing observations with frequency (%) for categorical variables.

Categorical variables were compared between groups (Robotic Assisted Surgery and Navigated Assisted) using the chi‐square test (or Fisher's exact test when necessary). Student's *t* test was used to compare the distribution of continuous variables (or Mann–Whitney's test when distribution departed from normality or when homoscedasticity is rejected). The analysis of factors independently associated with extended operative time was based on a logistic regression model including variables significantly associated with extended operative time in a univariate analysis (significance threshold of <0.20). The final model, comprising variables significantly (at a threshold of <0.05) and independently associated with extended time surgery, was obtained using a step‐by‐step, descending method. Intermediate nested models were compared using the likelihood ratio test. The goodness‐of‐fit of the model to the data was tested. All reported *p* values were two‐sided, and the significance threshold was <0.05. Statistical analyses were performed using EasyMedStat Version 3.40.

## RESULTS

Three hundred sixty‐seven patients were included in the study, with 122 being men (33.2%). The patients' median age was 78 years (50–93). There was no difference between the robot‐assisted and navigated TKA cohorts based on age, gender, BMI, side, ASA score, preoperative HKA, preoperative range of motion and approach. The baseline characteristics of the entire study cohort are shown in Table 1.

**Table 1:** Characteristics of the cohort.

	Navigation	Robot	*p*
	*n* = 218	*n* = 149	
Baseline characteristic			
Age (years), mean (range)	74.2 (50–93)	73.4 (51–93)	**0.366**
Male sex, no. (%)	74 (33.9%)	48 (32.2%)	0.818
Weight, kg (range)	76.8 (43–121)	75.8 (42–115)	0.508
Height, cm (range)	165 (147–190)	165 (145–190)	0.86
BMI, km/m^2^ (range)	28 (19.6–38.1)	27.5 (17.9–38)	0.366
Surgical parameter			
Side right, mean (%)	120 (55%)	72 (48.3)	**0.246**
HKA initial (°), mean (range)	176.3 (159–204)	174.7 (160–206)	0.144
Recurvatum (°), mean (range)	0.09 (0/10)	0.1 (0/5)	**0.643**
Flessum (°), mean (range)	2.9 (0/30)	3.4 (0/30)	**0.731**
Flexion (°), mean (range)	112 (80/140)	110 (60/140)	**0.288**
Approach sub/mid vastus, no (%)	137 (62.8%)	102 (68.4%)	**0.319**
CR (vs. PS), no (%)	100 (45.9%)	103 (69.1)	**<0.001**

*Note*: Bold values are statistically significant.

Abbreviation: BMI, body mass index; CR, cruciate‐retaining; HKA, hip–knee–ankle; PS, posterior‐stabilised.

### Learning curve with the use of robot

CUSUM analysis showed two stages: the initial learning curve corresponds to the number of cases needed for each surgeon to decrease their average operating time. Second, the plateau learning curve corresponds to the number of cases needed to become reproducible over its surgical time.

**Figure 3:**
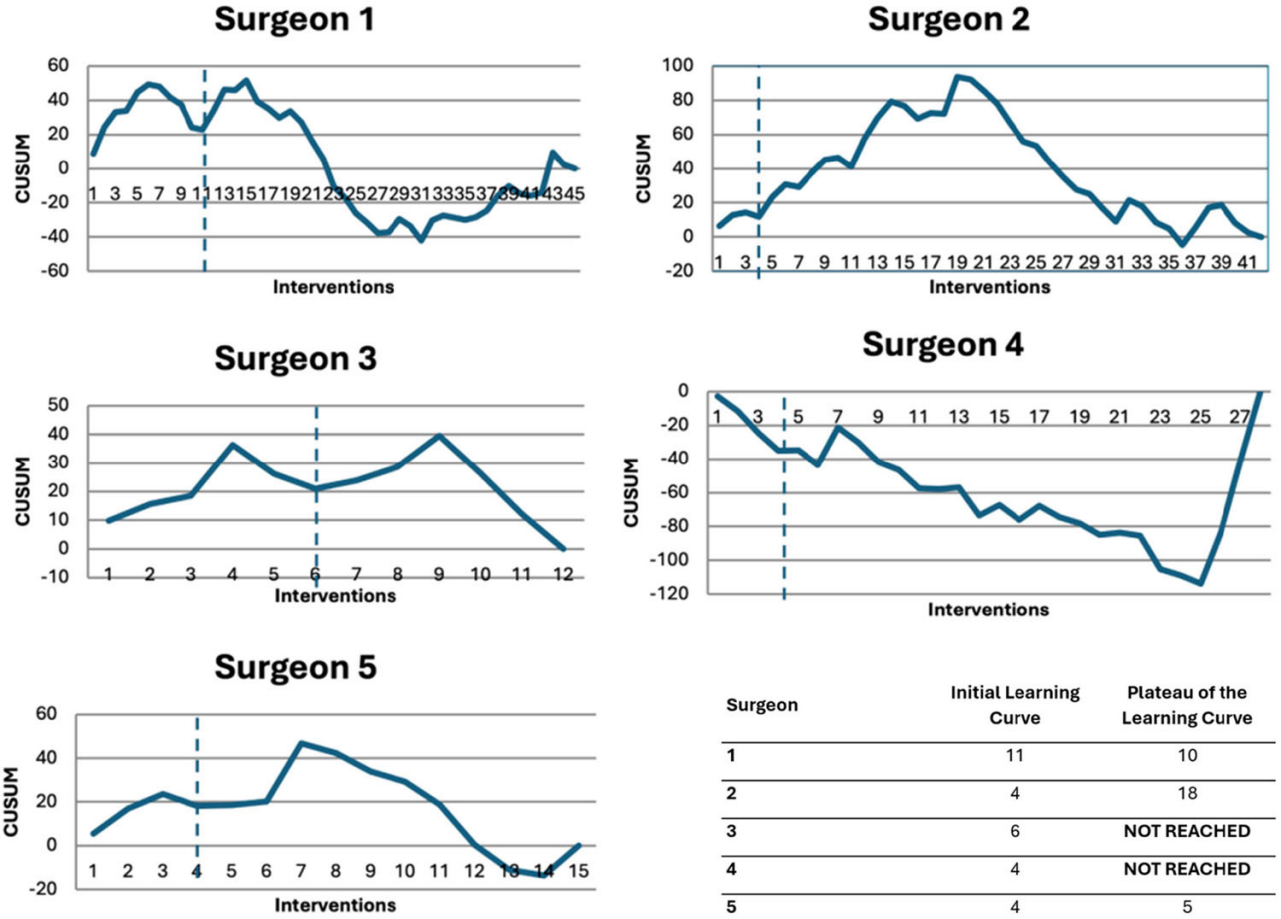
CUSUM analysis by the surgeon and its interpretation. CUSUM, cumulative summation.

CUSUM analysis has shown that the initial learning curve was reached for surgeons after performing between 4 and 11 cases (11 for surgeon no. 1, surgeon no. 4 for no. 2, surgeon no. 6 for no. 3, surgeon no. 4 for no. 4 and surgeon no. 4 for no. 5) (Figure 3).

Second, the plateau stage was only reached by three of the five surgeons (10 for surgeon no. 1, 18 for surgeon no. 2, 4 for surgeon no. 5 and not reached for surgeon nos. 3 and 4) (Figure 3).

### Operating time between navigation and robot

The robotic operating time was 57.1 min compared to 54.1 min (*p* = 0.017) with navigation, and the difference per surgeon was statistically significant only for the low‐volume surgeon (*p* = 0.008) (Table 2).

**Table 2:** Comparison of overall operating time and time per surgeon between robotic assistance and navigation.

	Navigation	Robot	*p*
	*n* = 218	*n* = 149	
Skin to skin (min), mean (range)	54.11 (28–125)	57.1 (32–101)	**0.017**
Operating time per surgeon			
No. 1, mean (SD)	60.63 (14.8)	61.4 (=−8.5)	0.076
No. 2, mean (SD)	41.7 (6.6)	43.2 (8.5)	0.556
No. 3, mean (SD)	69.8 (12.3)	79.7 (12.8)	0.008
No. 4, mean (SD)	51.9 (5.4)	55.4 (10)	0.155
No. 5, mean (SD)	56.4 (9.7)	62.4 (11.4)	0.058

*Note*: Bold value is statistically significant.

Abbreviation: SD, standard deviation.

### Factors associated with extended operative time

Multivariate analysis showed that the use of the robot (*p* < 0.001), surgeon (*p* < 0.001), use of a posterior‐stabilised implant (*p* < 0.001) and varus of more than 10° (*p* = 0.0107) were independently and significantly associated with extended operative time (Table 3).

**Table 3:** Multivariate analysis of the associated factors with extended operative time.

	Odds ratio	*p*
HKA initial ‎≤170°	3.05 [0.712–5.38]	**0.0107**
Use of robot	4.31 [2.17–6.45]	**<0.001**
PS (vs. CR)	5.74 [2.35–9.12]	**<0.001**
Surgeon reference: no. 2		
No. 1	17.71 [14.58–20.84]	<**0.001**
No. 3	28.79 [25.07–32.51]	<**0.001**
No. 4	6.39 [2.92–9.85]	<**0.001**
No. 5	15.27 [11.92–18.62]	<**0.001**

*Note*: Bold values are statistically significant.

Abbreviation: CR, cruciate‐retaining; HKA, hip–knee–ankle; PS, posterior‐stabilised.

## DISCUSSION

The most important finding of the present study was that we estimated a relatively short learning curve for several surgeons, switching from navigation assistance to robotics. Based on surgical time, surgeons need to perform between 4 and 11 cases to reach the initial learning curve. The main predictive factors for extended surgical time were the use of the robot (*p* < 0.001), surgeon (*p* < 0.001), posterior‐stabilised implants (*p* < 0.001) and varus of more than 10° (*p* = 0.0107).

### Learning curve with the use of robot

Concerning the first aim, these results are consistent with the literature, with a relatively short initial learning curve, calculated to be between 4 and 11 cases. Previous publications have shown that, depending on the type of assistance used, surgeons require between 2 and 15 robotic instrument insertions to achieve similar operating times as those with well‐trained traditional methods [8, 16, 18, 27, 30]. Looking only at the same device in the literature, robotic surgery times were reported to be equal to traditional instrumentation between two and nine robotic surgeries [16, 18]. In contrast to our study, these publications are based on the experience of a single surgeon, which limits their generalisability to the wider population of arthroplasty surgeons, as the specific skills and aptitude of one surgeon for robotic methods may differ from those of other surgeons [16].

One potential factor contributing to the observed differences in learning curves among the surgeons may be the staggered adoption of robotic assistance. Surgeon 1, who exhibited the longest learning curve, began using the robot approximately three months before the others. This earlier start could have influenced the outcomes, particularly given that all surgeons worked with the same paramedical teams. Within the team, surgical practices and techniques were regularly discussed, and advice was shared among colleagues. As a result, it is possible that the surgeons who started later may have benefited from a more experienced and better‐prepared support team, as well as insights gained from their peers' earlier experiences.

One of the strengths of this study is that we estimated not only the initial learning curve, which is the most reported in the studies [16], but also the plateau learning curve. The learning curves of surgeons demonstrate considerable variability. For Surgeons 1 and 5, the initial and plateau learning curves indicate steady progress and early stabilisation. Surgeon 2 displays a discrepancy between the two stages, suggesting initial improvement followed by persistent variability before actual stabilisation. This may be due to atypical early cases or changes in the surgical context. With such a small number of patients, the variable difficulty of the cases is a limitation of the CUSUM analysis method. This may also explain why this surgeon's operating time drops drastically after 20 cases. For low‐volume surgeons (numbers 4 and 5), the absence of stabilisation in the operative time may reflect ongoing variability, a longer learning curve, or a lack of stabilisation during the study period. If they demonstrate a relatively short initial learning curve, the plateau learning curve has not been reached. Long‐term stabilisation and reproducibility of results tend to require more time. This may be attributed to reduced surgical activity and, more importantly, to the extended intervals between procedures, which limit consolidation of technical skills and procedural consistency over time. Additionally, variability in the complexity of the initial cases performed by low‐volume surgeons cannot be ruled out. For surgeon no. 4, the progressive increase in operative times over the study period is unexpected. A plausible explanation, beyond potential variability in case complexity, is the initial presence of a company representative in the operating room. This external support may have temporarily enhanced performance during the early cases, similar to the effect of having an experienced assistant, thereby artificially accelerating the initial phase of the learning curve [22].

### Operating time between navigation and robot

Overall, in this study, robotic assistance was associated with a significantly longer operative time compared to navigation (*p* = 0.017). However, this finding must be interpreted considering the surgeon's experience. When comparing mean operative times between navigated and robotic procedures, a statistically significant difference was observed only for one low‐volume surgeon (*p* = 0.008). In addition, it is noteworthy that, for one of the highest‐volume surgeons, the use of robotics improved the reproducibility of operative time, as evidenced by a reduced standard deviation compared to navigated surgery.

Therefore, the difference in operative time should be interpreted with caution, especially since some studies have reported shorter durations with robotic assistance compared to navigation [4, 10]. In the present analysis, the average difference was only 3 min per case, a duration unlikely to have a substantial impact on the overall schedule of a surgical day. This time interval remains well below the established 15‐min threshold identified as an independent risk factor for the development of postoperative infection [17].

### Associated factors with extended operating time

The second aim of our study was to identify factors associated with extended operative times.

The identity of the surgeon was identified as an independent factor associated with increased operative time (*p* < 0.001). This finding appears consistent with differences in surgical experience and annual knee arthroplasty volume among the team members. While some surgeons had a dedicated focus on prosthetic surgery, others had a broader practice profile, including foot surgery and trauma. This aligns with studies that do not report prolonged operative times following the adoption of robotic assistance among high‐volume surgeons [4, 10].

Consistent with the literature, the use of a posterior‐stabilised implant (p < 0.001) was associated with extended operating time [15, 29]. Among the explanations, to implant a posterior‐stabilised implant, we have to temporarily fix a notch guide to perform the notch cut, which is longer to perform than the sulcus femoral cut for a cruciate‐retaining implant. Posterior‐stabilised implants are more constrained compared to the cruciate‐retaining implant [26]. In their daily practice, the surgeons preferentially use this kind of implant for major deformation, which was also associated with major operating time (*p* = 0.0107). However, it is surprising that this difference was not found in extreme valgus. This may be due to a lack of power, as the cohort had fewer extreme valgus knees than extreme varus knees.

Finally, after multivariate analysis, robotic assistance was still associated with an extended operative time (*p* < 0.001). Further researches are needed to determine whether, with more experience, it could shorten operating time for high‐volume surgeons as reported in the literature [4, 10, 18]. As reported in other studies, the robot could be associated with long‐term savings by reducing the surgical complication rate and the need for follow‐up care in the short term by reducing operating time for high‐volume surgeons [3, 13]. This is especially true as an early outcome study suggests an improvement in knee function and pain with activity at discharge and six weeks [5]. Moreover, the reduced complication rate in RA‐TKA, without increased costs, supports its increased utilisation. This challenges the notion that medical innovation always leads to higher costs [13]. While the initial costs of RA‐TKA may be substantial, the long‐term economic benefits due to reduced complications can be significant, especially in high‐volume settings.

### Limitations

Several limitations should be outlined in this study. It was a non‐randomised, retrospective, descriptive study involving patients from a single centre. If the evaluation of the practice of a team of five surgeons is a strength, it must be emphasised that the number of cases per surgeon was uneven, although this did not affect the evaluation of a learning curve based on operative time. It can explain why not all surgeons reach the plateau of the learning curve.

This publication is the only one that has studied a team of surgeons who have only changed the arthroplasty assistance without changing their alignment philosophy. Therefore, these results cannot be generalised to surgeons who, in addition to using robotic assistance, wish to move from mechanical alignment to personalised alignment. Finally, the absence of clinical and radiological outcome assessment is a limitation of the current study and may serve as a valuable focus for future investigations.

## CONCLUSION

In this study, the learning curve associated with VELYS™ was relatively short, between 4 and 11 procedures. The small increase in operative time compared to navigation should not be a barrier to the adoption of robotic assistance.

## AUTHOR CONTRIBUTIONS

All authors contributed to the creation of this study. Designed the study, interpreted the data, wrote and reviewed the manuscript: Alexandre Le Guen. Designed the study and reviewed the manuscript: Antoine Mouton, Guillaume Auberger, Vincent Le Strat and Simon Marmor. Designed the study, contributed to the data analysis, supervised the study, edited and reviewed the manuscript: Thomas Aubert. All authors have read the final manuscript and approved it for publication.

## CONFLICT OF INTEREST STATEMENT

Antoine Mouton serves as a consultant for Corin, Amplitude, ConMed and Johnson & Johnson and receives royalties from ConMed. Simon Marmor serves as a consultant for Amplitude and Johnson & Johnson and receives royalties from Amplitude. Thomas Aubert is a consultant for Corin and Johnson & Johnson. The remaining authors declare no conflicts of interest.

## ETHICS STATEMENT

Referenced under CNIL MR004 2225508. The study was submitted to the Ethics Committee for Clinical Research of Groupe Hospitalier Diaconesses Croix Saint‐Simon on 13 January 2025 and received a favourable opinion on 27 January 2025. The committee confirms that the study complies with generally accepted scientific principles, ethical standards and current regulations. Informed consent was obtained from all patients.

## Data Availability

Data are available upon reasonable request.
